# Grapheme-color synaesthesia is associated with a distinct cognitive style

**DOI:** 10.3389/fpsyg.2013.00632

**Published:** 2013-09-19

**Authors:** Beat Meier, Nicolas Rothen

**Affiliations:** ^1^Department of Psychology, Institute of Psychology and Center for Cognition, Learning and Memory, University of BernBern, Switzerland; ^2^Department of Psychology and Sackler Centre for Consciousness Science, University of SussexBrighton, UK

**Keywords:** visualizer, verbalizer, imagery, learning style, memory, creativity

## Abstract

In this study we investigated whether synaesthesia is associated with a particular cognitive style. Cognitive style refers to preferred modes of information processing, such as a verbal style or a visual style. We reasoned that related to the enriched world of experiences created by synaesthesia, its association with enhanced verbal and visual memory, higher imagery and creativity, synaesthetes might show enhanced preference for a verbal as well as for a visual cognitive style compared to non-synaesthetes. In Study 1 we tested a large convenience sample of 1046 participants, who classified themselves as grapheme-color, sound-color, lexical-gustatory, sequence-space, or as non-synaesthetes. To assess cognitive style, we used the revised verbalizer-visualizer questionnaire (VVQ), which involves three independent cognitive style dimensions (verbal style, visual-spatial style, and vivid imagery style). The most important result was that those who reported grapheme-color synaesthesia showed higher ratings on the verbal and vivid imagery style dimensions, but not on the visual-spatial style dimension. In Study 2 we replicated this finding in a laboratory study involving 24 grapheme-color synaesthetes with objectively confirmed synaesthesia and a closely matched control group. Our results indicate that grapheme-color synaesthetes prefer both a verbal and a specific visual cognitive style. We suggest that this enhanced preference, probably together with the greater ease to switch between a verbal and a vivid visual imagery style, may be related to cognitive advantages associated with grapheme color synaesthesia such as enhanced memory performance and creativity.

## Introduction

Synaesthesia involves unusual experiences, such as color experiences in response to letters printed in black (grapheme-color synaesthesia), color experiences in response to music and sound (sound-color synaesthesia), gustatory experiences in response to words and names (lexical gustatory synaesthesia), or the activation of a spatial representation when confronted with an element that belongs to a particular sequence, for example, a day of the week (sequence-space synaesthesia). As synaesthesia—even different types of it—runs in families, it is assumed to have a genetic component (Barnett et al., 2008a; Asher et al., 2009) and, in fact, frequently different types of synaesthesia co-occur within the same individual (Rich et al., 2005; Simner et al., 2006; Novich et al., 2011). Evidence from neuroscience indicates that synaesthesia is associated with structural brain differences such as increased local and global connectivity (Rouw and Scholte, 2007; Hanggi et al., 2011; Rouw et al., 2011). Moreover, for grapheme-color synaesthesia, there is evidence that it is associated with basic differences in perception and excitability of the visual cortex (Barnett et al., 2008b; Terhune et al., 2011). Synaesthesia has cognitive consequences such as enhanced memory performance (Smilek et al., 2002; Rothen et al., 2012; Meier and Rothen, 2013), and greater involvement in creative activities (Rich et al., 2005; Ward et al., 2008; Rothen and Meier, 2010b). Moreover recent evidence suggests that synaesthesia is associated with an atypical personality profile (Banissy et al., 2012, 2013). Here we investigate whether synaesthesia is associated with a distinct cognitive style.

Cognitive style refers to a preference for processing information in a certain manner, thus representing consistencies in cognitive functioning of a person, particularly with respect to acquiring and processing information (Blazhenkova and Kozhevnikov, 2009). An important distinction is between verbalizers and visualizers (Paivio, 1971; Richardson, 1977), with the former assumed to rely more on verbal strategies and the latter more on visual strategies when performing a cognitive task. Originally, this was thought to be a unidimensional construct with verbal and visual preference as complementary poles. However, subsequent research has shown that verbal and visual style dimensions are independent. Moreover, both psychometric and neuroscientific evidence has revealed that the visual factor can be further subdivided into two independent visual style dimensions: a spatial imagery style and an object imagery style, the former corresponding to the dorsal and the latter to the ventral system of visual information processing (Kozhevnikov et al., 2005; Blazhenkova and Kozhevnikov, 2009; Borst et al., 2011). This distinction is also reflected in one of the most common self-report measures, the revised Verbalizer-Visualizer Questionnaire (VVQ), in which the spatial visualizer style was complemented with a vividness of imagery style (Kirby et al., 1988), reflecting the fact that object visualizers are especially good in generating vivid pictorial images.

Neuroimaging evidence shows that cognitive style affects individual differences in domain-specific brain activations (Kraemer et al., 2009; Hsu et al., 2011). Using functional magnetic resonance imaging (fMRI), Kraemer et al. (2009) compared correlations between brain activations during a word-based and a picture-based task with verbalizer and visualizer scores of the revised VVQ. They found that for the picture-based task, the verbalizer ratings correlated with brain activity in the area of the left supramarginal gyrus. In contrast, in the word-based task, visualizer ratings correlated with brain activity in the right fusiform gyrus. Thus, higher scores on the verbal style dimension were associated with activations in phonological areas when processing pictorial representations and higher scores on the visual style dimension were associated with activations in the visual cortex when processing written descriptions of visual features. These results suggest that a particular cognitive style activates style-specific brain areas, particularly when the information is not presented in that domain.

In a follow-up study, Hsu et al. (2011) extended these results with a verbal task during which participants were required to retrieve more or less detailed color information. They found more activation in the left fusiform gyrus during retrieval of more detailed color knowledge and this activation correlated with a visual style preference, again suggesting that cognitive style was associated with processing information in the style-specific mode.

It is noteworthy that the brain areas in inferior temporal and inferior parietal regions which are associated with a particular cognitive style, are often found to be more activated in grapheme-color synaesthesia in response to achromatic graphemes (Nunn et al., 2002; Hubbard and Ramachandran, 2005; van Leeuwen et al., 2010). Thus, rather than showing a preference for either a verbal or a visual cognitive style, it is possible that synaesthetes have higher scores on both verbalizer and visualizer style dimensions. Consistent with this notion is the fact that grapheme-color synaesthesia is associated with enhanced verbal *and* visual memory, higher imagery and creativity, and more generally, an enriched world of experiences (Meier and Rothen, 2013). This may go together with enhanced preference for a verbal *and* a visual cognitive style and the goal of this study was to investigate this possibility.

In Study 1 we used a convenience sample of more than thousand participants who filled out a brief survey on our synaesthesia research webpage. In Study 2, we specifically tested grapheme-color synaesthetes with confirmed grapheme-color consistency in a laboratory study.

## Study 1

### Method

#### Participants

The sample consisted of participants who filled out the Synaesthesia-Check on the synaesthesia research webpage of the University of Bern (www.synaesthesie.unibe.ch). The Synaesthesia-Check is a short questionnaire used to establish contact with the general public interested in our research. It involves questions about potential forms of synaesthesia such as grapheme-color, sound color, lexical gustatory, and sequence space, demographic information, and it provides the opportunity to leave contact information for those willing to take part in future studies.

In this study we report data that were collected in the period from October 2010 until April 2013. From a total of 3320 people who completed the Synaesthesia-Check, 1046 also agreed to fill out a questionnaire on cognitive style that was then appended at the end of the Synaesthesia-Check. The supplied mean age of these participants was 30.9 years (*SD* = 13.14), 79% of them females, and 88% of them right-handed. Overall, 83% of these participants indicated to have at least one of the four forms of synaesthesia. The exact number of participants for each form and each combination of different forms is presented in Table 1.

**Table 1:** **Descriptive statistics for cognitive style measures in Study 1**.

		**Verbalizer**		**Spatial visualizer**		**Vivid imagery visualizer**	
	***N***	**Mean**	***SE***	**Mean**	***SE***	**Mean**	***SE***
GC SC LG SS	85	6.59	0.57	7.92	0.56	12.79	0.59
GC SC LG	44	7.55	0.82	7.73	0.85	12.23	0.96
GC SC SS	93	5.72	0.58	7.57	0.61	12.22	0.59
GC SC	101	5.26	0.52	7.82	0.58	10.92	0.62
GC LG SS	22	5.68	1.64	6.14	0.97	12.27	1.11
GC LG	24	7.42	1.19	6.75	0.88	12.38	1.08
GC SS	64	5.81	0.81	7.75	0.64	12.27	0.60
GC	196	5.62	0.41	7.05	0.40	9.35	0.44
SC LG SS	18	6.89	1.23	7.89	1.27	13.33	0.83
SC LG	26	5.88	1.42	7.58	1.22	11.35	1.11
SC SS	27	4.81	1.12	7.15	1.06	9.48	1.15
SC	55	4.85	0.92	7.44	0.83	12.58	0.63
LG SS	21	2.43	1.28	9.33	1.33	10.62	1.51
LG	42	4.95	0.99	5.88	1.00	7.86	1.07
SS	51	5.12	0.89	6.78	0.75	8.29	0.96
CG	177	4.40	0.48	7.07	0.44	6.97	0.55

GC, Grapheme Color; SC, Sound Color; LG, Lexical Gustatory; SS, Sequence Space; CG, Control Group (no synaesthesia).

#### Material and procedure

In order to assess cognitive style, we used the 30-items revision of the VVQ Verbalizer-Visualizer-Questionnaire (Richardson, 1977; Kirby et al., 1988). The VVQ consists of three scales, verbal style, visual style, and vivid imagery style, each with appropriate psychometric properties (Kirby et al., 1988). A high score on the verbal dimension of the questionnaire is associated with a preference for verbal representations and an enhanced ability to work with verbal materials (e.g. “I enjoy doing work that requires the use of words”). A high score on the visual dimension of the questionnaire is associated with a preference for visuo-spatial representation formats and with the ability to imagine spatial compositions of scenes or real world objects (e.g., “When I read books with maps in them, I refer to the maps a lot”). A high score on the vivid imagery style is associated with a preference to let the mind wander and the ability to generate vivid mental images, particularly related to dream imagery (e.g., “My dreams are sometimes so vivid I feel as though I actually experience the scene”). Each scale consists of 10 statements, five worded positively and five worded negatively. Participants had to rate each statement on a discrete five-point scale, from strongly agree (5) to strongly disagree (1). Scores for each scale were summed separately. The five positively worded questions received positive scores and the five negatively worded questions received negative scores. Thus, scores from −20 to 20 were possible. Data collection was administered on-line via the internet and lasted approximately 10 min, with text appearing in black on a white background.

#### Design

Given that different types of synaesthesia often co-occur and our large data-set, we decided to use analyses of variance (ANOVA) with the four types of synaesthesia (grapheme-color, sound-color, lexical-gustatory, and sequence-space) as factors, each with two levels (i.e., present/absent). Hence, it was possible for a given individual to have several types (i.e., multiple synaesthetes) or no synaesthesia at all (i.e., control group). This design allowed to identify types of synaesthesia that influence cognitive style independently and it also allowed to detect interaction effects (e.g., enhancing effects from the presence of multiple types of synaesthesia). As dependent variable, each of the three cognitive style dimensions was analyzed separately. To determine significance an alpha level of 0.05 was used.

### Results

Table 1 presents descriptive information for verbalizer, spatial visualizer, and vivid imagery visualizer styles for each factorial combination of the four types of synaesthesia and the control group, separately.

**Figure 1:**
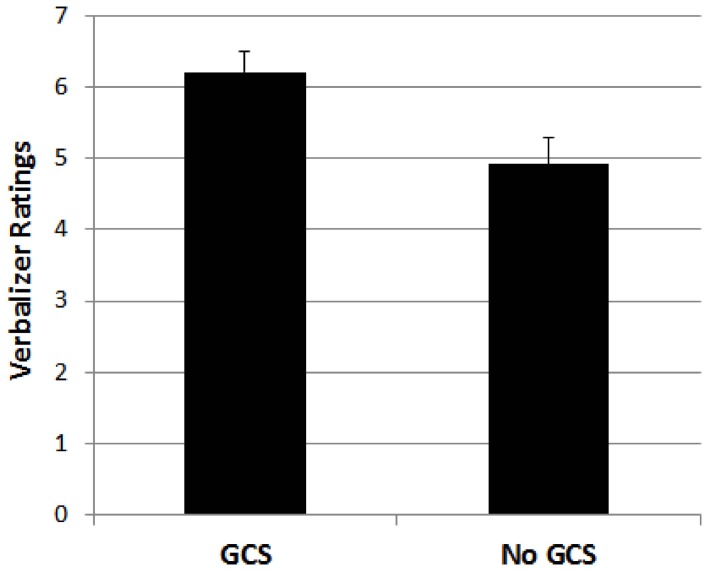
**Mean ratings for participants with grapheme-color synaesthesia (GCS) and without grapheme-color synaesthesia (No GCS) on the verbal style dimension**. Error bars represent standard errors.

For the verbalizer style dimension, a 4-factorial ANOVA with the four types of synaesthesia as binary factors revealed a significant effect of grapheme-color synaesthesia, *F*_(1, 1030)_ = 7.195, *p* < 0.01, indicating higher ratings for participants who indicated to have grapheme-color synaesthesia compared to those who did not indicate to have grapheme-color synaesthesia. This result is depicted in Figure 1. No other effect reached significance, all *F*s < 3.1, *p*s > 0.08.

For the spatial visualizer style dimension, the same kind of analysis revealed no significant effects, neither differences between the four types of synaesthesia, nor any interaction effect, all *F*s < 2.1, *p*s > 0.15.

For the vivid imagery visualizer style dimension, the 4-factorial ANOVA showed significant main effects, with *F*_(1, 1030)_ = 12.56, *p* < 0.01, for grapheme-color synaesthesia, *F*_(1, 1030)_ = 14.36, *p* < 0.01, for sound-color synaesthesia, *F*_(1, 1030)_ = 7.47, *p* < 0.01, and for lexical-gustatory synaesthesia, and a marginally significant effect for sequence-space synaesthesia, *F*_(1, 1030)_ = 3.79, *p* = 0.052. Moreover, there was a double interaction between grapheme-color and sound-color synaesthesia, *F*_(1, 1030)_ = 7.99, *p* < 0.01, as well as a triple interaction between grapheme-color synaesthesia, lexical-gustatory synaesthesia and sequence-space synaesthesia, *F*_(1, 1030)_ = 6.83, *p* < 0.01. No other effect reached significance, all *F*s < 2.1, *p*s > 0.15.

**Figure 2:**
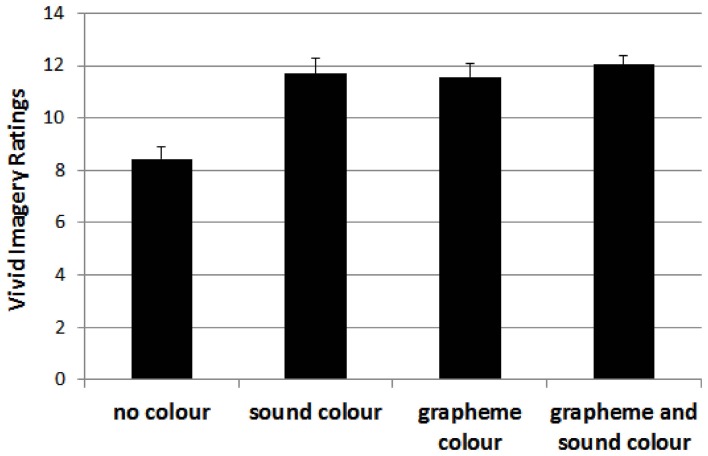
**Mean ratings for participants without color synaesthesia (no color) and those with color synaesthesia (i.e., sound-color, grapheme-color or both) on the vivid imagery style dimension**. Error bars represent standard errors.

The double interaction is depicted in Figure 2. It indicates that the presence of a color synaesthesia (i.e., either grapheme-color, or sound-color, or both) is critical for the higher vivid imagery visual style ratings. However, it also indicates that the presence of both kinds of color synaesthesia's did not further boost the vivid imagery visual style ratings.

**Figure 3:**
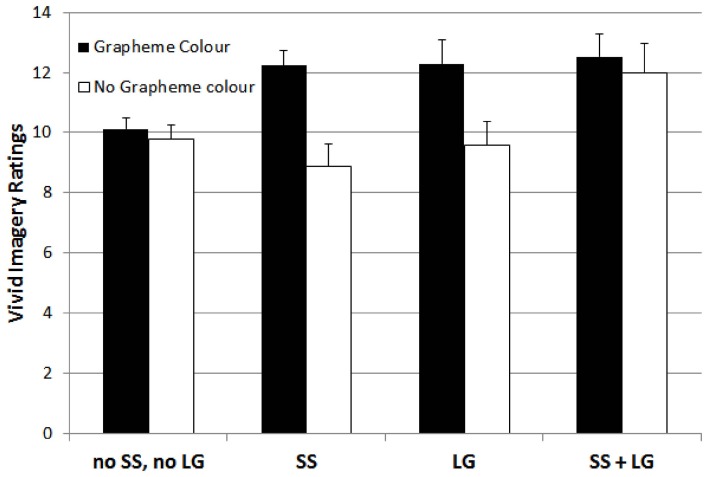
**Mean ratings for participants with neither sequence-space nor lexical gustatory synaesthesia (no SS, no LG), sequence-space synaesthesia (SS), lexical gustatory synaesthesia (LG), and with both sequence-space and lexical gustatory synaesthesia (SS + LG), separated by whether or not grapheme-color synaesthesia was also present, on the vivid imagery style dimension**. Error bars represent standard errors.

The triple interaction is depicted in Figure 3. It indicates that in the absence of grapheme-color synaesthesia, the presence of both, lexical-gustatory synaesthesia and sequence-space synaesthesia also enhanced the vivid imagery visualizer style ratings. However, the presence of only either sequence-space or lexical-gustatory synaesthesia was not sufficient to boost the vivid imagery visualizer style ratings in the absence of grapheme-color synaesthesia.

In order to investigate the independence of the three cognitive style dimensions, we also performed correlational analyses. The correlations between verbal and spatial visualizer style, verbal and vivid imagery visualizer style, and spatial visualizer and vivid imagery visualizer style were *r* = −0.08, *r* = 0.18, and *r* = 0.17 (all *p*s < 0.01), respectively, representing low effect sizes, and replicating the substantial independence of the three style dimensions (Kirby et al., 1988).

### Discussion

The results of Study 1 show a clear association between synaesthesia and a distinct pattern of cognitive style. Specifically, grapheme-color synaesthetes scored higher on the verbal style factor. Moreover, several types of synaesthesia go together with a more vivid imagery style. Importantly, the results suggest that those types of synaesthesia which invoke color have the most pronounced effect. In the absence of colored synaesthesia, the presence of multiple types of non-colored synaesthesia such as lexical-gustatory together with sequence space also enhanced a vivid imagery visualizer style. In contrast, the visualizer style dimension that is more concerned with spatial relations was not affected by the presence of any kind of synaesthesia at all, not even sequence-space synaesthesia (cf., Price, 2009, for a similar result for spatial imagery).

Although the pattern of these results is suggestive, we acknowledge that group membership was self-referred and we did not have any objective measurement to confirm the genuineness of synaesthesia. Thus, a replication under more controlled conditions is warranted. In Study 2 we used a laboratory setting and we administered a test of consistency to objectify the presence of synaesthesia (Eagleman et al., 2007).

## Study 2

### Method

#### Participants

For Study 2, a group of 24 grapheme-color synaesthetes and a control group consisting of 48 non-synaesthetes matched for age (Synaesthetes: *M* = 35.5 years, *SD* = 14.9; Control group: *M* = 35.8, *SD* = 14.8), gender (Synaesthetes: 22 females, Control group: 44 females) and handedness (Synaesthetes: 22 right-handed, Control group: 44 right-handed) participated. None of them had already participated in Study 1. For each synaesthete, two control subjects were yoke-matched. Inclusion criterion for the synaesthetes was the presence of grapheme-color synaesthesia. We also asked them about the presence of other forms of synaesthesia. It turned out that five of them also reported to have sequence-space synaesthesia. None reported to have sound-color or lexical gustatory synaesthesia.

#### Material and procedure

Participants were tested in the laboratory and we administered a test of consistency to verify the grapheme-color synaesthesia, for which grapheme-color associations were assessed by means of a computerized color palette (Meier and Rothen, 2007). RGB-values were converted into CIELUV-values and Euclidian distances were calculated as consistency scores, with smaller values indicating higher consistency (Rothen et al., 2013). For synaesthetes, mean distance in CIELUV space was 27.5 (*SD* = 13.3), and for the control group, it was 103.4 (*SD* = 29.1). These consistency scores were significantly different, *t*_(70)_ = 12.1, *p* < 0.001, thus confirming the higher consistency of the synaesthetes.

We used the same VVQ-questionnaire as in Study 1 to assess the three cognitive style dimensions, but this time a paper/pencil version was administered. The VVQ was administered as part of a larger test battery which lasted about two and a half hours. The consistency test was conducted at the beginning and at the end of the test session.

### Results

**Figure 4:**
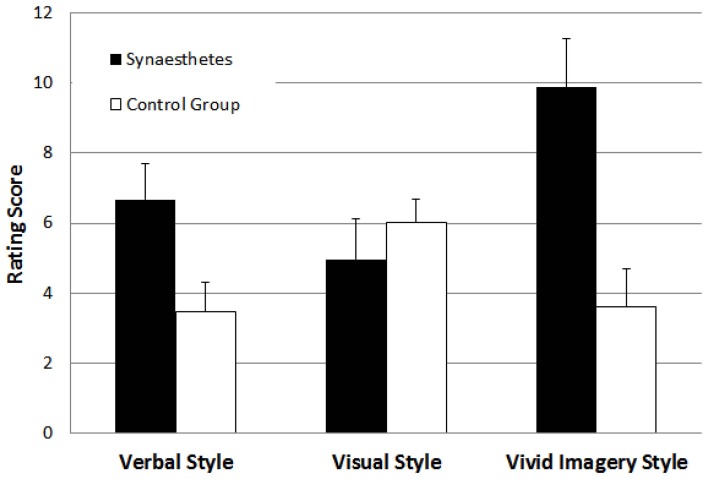
**Mean ratings for participants with and without grapheme-color synaesthesia on the verbal style, the visual style, and the vivid imagery cognitive style dimensions in Study 2**. Error bars represent standard errors.

The results of each of the three cognitive style ratings are shown in Figure 4, separately for synaesthetes and the non-synaesthete control group. To test for group differences, we used independent *t*-tests for each cognitive style dimension separately. For the verbal style dimension, the grapheme-color synaesthetes scored higher than the control group, *t*_(70)_ = 2.36, *p* < 0.05. For the spatial visualizer style dimension, there was no difference between groups, *t*_(70)_ = 0.86, *p* > 0.05. For the vivid imagery visualizer style dimension, the grapheme-color synaesthetes scored higher than the control group, *t*_(70)_ = 3.43, *p* < 0.05. Thus, these results replicate the main results from Study 1 in a controlled laboratory setting.

As in Study 1, we used correlational analyses to investigate the independence of the three cognitive style dimensions. The correlations between verbal and spatial visualizer style, verbal and vivid imagery visualizer style, and spatial visualizer and vivid imagery visualizer style were *r* = −0.05 (*p* = 0.69), *r* = 0.26 (*p* < 0.05), and *r* = −0.16 (*p* = 0.19), respectively, representing low effect sizes, and indicating again the substantial independence of the three style dimensions.

### Discussion

The results of Study 2 replicate the main results of the first study by demonstrating again enhanced ratings for a verbal cognitive style and a vivid imagery style, but no differences for spatial visual style. A comparison with Study 1 shows that the scores of the verbalizer scale were very similar. However, for the spatial visualizer style and the vivid imagery style the score was somewhat smaller. We have no straight-forward explanation for this discrepancy. One possibility is that the change in the test format may have affected the scoring on the visual scales. Another possibility is that as different groups were tested this may simply reflect random sampling variation. Importantly, however, the general pattern of results was replicated and it occurred within a sample with confirmed grapheme-color synaesthesia as indicated by a test of consistency, and a tightly matched control group under well-controlled laboratory conditions. This indicates that grapheme-color synaesthesia is associated with a distinct cognitive style. In the General Discussion we elaborate on the broader implications of these results.

## General discussion

This study provides the first systematic investigation into the relationship between cognitive style and synaesthesia. Inspired by the findings related to the cognitive consequences of synaesthesia for memory, creativity, and imagery, and by findings from neuroimaging studies, we hypothesized that in synaesthesia a preference for both verbal and visual cognitive styles may be prevalent. The results of two studies confirm this assumption. Particularly, we found that grapheme-color synaesthesia is associated with a preference for both a verbal style and for a vivid imagery style. Further results from Study 1 suggest that a preference for a more vivid imagery style may be prevalent also for other forms of synaesthesia, in particular those that involve color (i.e., grapheme-color and sound-color), and in the case of the absence of grapheme-color synaesthesia, those that involve multiple other forms such as lexical-gustatory and sequence-space. Thus, in the absence of color synaesthesia, the presence of multiple forms of synaesthesia may also induce a richer world of experience that is then sufficient to enhance the preference for a vivid imagery cognitive style.

The result of enhanced verbalizer style in grapheme-color synaesthesia is consistent with the idea that synaesthetes have a particular affinity for information that is related to their inducer, that is, verbal materials in the case of grapheme-color synaesthesia. This affinity may have consequences in other cognitive domains such as enhanced memory for words and more generally, the verbal domain (Rothen and Meier, 2010a). Moreover, there are many famous writers such as Nabokov, Baudelaire, or Wittgenstein who were also synaesthetes and it is possible that there is a link between synaesthesia and a preference for verbal processing. Recently, Banissy et al. (2013) found that grapheme-color synaesthesia was associated with higher scores in the *openness to experience* personality factor, which is correlated with verbal intelligence (Ziegler et al., 2012). Notably, in grapheme-color synaesthesia the inducers are instances of sequences such as digits, letters, or days of the week, and processing such sequences may involve mainly serial, analytic processing tuning the cognitive system for a verbal cognitive style.

The result of enhanced vivid imagery visualizer style in synaesthesia is also consistent with the idea that synaesthetes have a particular affinity for information that is related to their concurrent, that is, visual and particularly color information in the case of grapheme-color synaesthesia. This affinity may also have consequences in other cognitive domains such as enhanced perception for colors, enhanced memory for colors (Yaro and Ward, 2007; Pritchard et al., 2013; Terhune et al., 2013) and more generally, the visual domain (Rothen and Meier, 2010a).

The result of a more vivid imagery visualizer style in synaesthetes is also consistent with reports of atypical experiences in color synaesthetes, such as more strange perceptual experiences and hallucinations and more fantasizing (Banissy et al., 2012, 2013). Moreover, an enhanced vivid imagery visualizer style can be considered to be directly related to findings from studies that investigated imagery abilities in synaesthetes and non-synaesthetes (Barnett and Newell, 2008; Spiller and Jansari, 2008; Price, 2009). Using an experimental procedure, Spiller and Jansari (2008) found that grapheme-color synaesthetes were faster than a matched control group in an imagery task that involved the generation and inspection of visual images. Using a self-report measure, Barnett and Newell (2008) found that a group of synaesthetes reported experiencing more vivid mental images than controls. Price (2009) also found that people with sequence space synaesthesia reported more vivid mental imagery. In contrast, no such difference was evident for spatial imagery.

The absence of an advantage in spatial imagery in sequence-space synaesthesia is consistent with our findings from the spatial visualizer style, in which we did not find any differences between synaesthetes and non-synaesthetes. There is evidence that a spatial visualizer style is pre-dominant in scientists such as physicists and engineers, while a vivid imagery visualizer style is more pre-dominant in visual artist such as professional painters, photographers and interior designers (Kozhevnikov et al., 2005). Given that synaesthetes have a propensity to engage in creative activities and occupations more than in science, it may not be surprising that we did not find any differences on the spatial visualizer style.

Our results suggest that a preference for both a verbal and a vivid imagery visual style can co-occur within the same group of individuals and that this distinct pattern is associated with grapheme-color synaesthesia. As noted above, graphemes are instances of sequences which involve serial, analytic processing, favoring a verbal cognitive style. However, the concurrents are visual instances, most often colors and visual forms, which involve parallel or holistic processing, thus favoring a visual cognitive style. The combination of a verbal and a vivid imagery visual style in grapheme-color synaesthetes and their propensity to switch easily between these styles may be related to the cognitive benefits associated with grapheme-color synaesthesia. On the other hand, it also might be the result of their better cognitive performance in this domain so they will adapt a cognitive style that fits their ability. Further research is necessary to determine the causality of this relationship.

We have proposed elsewhere that synaesthetic concurrents are represented as additional features in the semantic network, and via spreading activation they allow for supplementary connections between concepts (Rothen et al., 2010; Meier, in press). Here we would like to emphasize that a core difference between synaesthetes and non-synaesthetes may not only be that more connections between semantic representations are existing in the synaesthete brain, but that also the way these representations can be accessed may differ. That is, due to an affinity toward both verbalizer and visualizer processing styles multiple pathways are available to access these representations. In light of these considerations the observed differences between synaesthetes and non-synaesthetes in memory and creativity can be easily accounted for. There is converging evidence that a synaesthete's brain is in fact hyperconnected, not only locally in the fusiform gyrus and the inferior parietal lobe, but also globally (Hanggi et al., 2011). Nevertheless it is not clear whether the hyperconnected brain is the cause of synaesthesia or the consequence. It is possible that due to the lifelong associations between synaesthetic inducers and concurrents together with the propensity to favor multiple cognitive styles the hyperconnected brain in synaesthesia represents a remarkable example of brain plasticity (Bargary and Mitchell, 2008; Cohen Kadosh and Walsh, 2008; Hubbard et al., 2011; Rouw et al., 2011).

To summarize, in the present study, we show that grapheme-color synaesthesia is associated with a distinct cognitive style, namely a preference for both a verbalizer style and a vivid imagery visualizer style. Moreover, our results suggest that, at least a vivid imagery visualizer style may also be more prevalent in other forms of synaesthesia. It is an interesting direction for future research to investigate cognitive styles differences in these types of synaesthesia in more detail.

### Conflict of interest statement

The authors declare that the research was conducted in the absence of any commercial or financial relationships that could be construed as a potential conflict of interest.
